# Supplementary material for: Copper (I) or (II) Replacement of the Structural Zinc Ion in the Prokaryotic Zinc Finger Ros Does Not Result in a Functional Domain
Source: Int J Mol Sci. 2022 Sep 20;23(19):11010. doi: 10.3390/ijms231911010 (PMC9569694; doi:10.3390/ijms231911010)
Supplement: Supplementary file 1 [file ijms-23-11010-s001.zip › ijms-1858000-supplementary.pdf]

Copper (I) or (II) Replacement of the Structural Zinc Ion in the Prokaryotic Zinc Finger Ros Does Not Result in a Functional Domain

Martina Dragone [1], Rinaldo Grazioso [1], Gianluca D'Abrosca [1], Ilaria Baglivo [1], Rosa Iacovino [1], Sabrina Esposito [1], Antonella Paladino [2], Paolo V. Pedone [1], Luigi Russo [1], Roberto Fattorusso [1], Gaetano Malgieri [1] and Carla Isernia [1,*]

[1] Department of Environmental, Biological and Pharmaceutical Science and Technology, University of Campania "Luigi Vanvitelli", Via Vivaldi 43, 81100 Caserta, Italy
[2] Institute of Biostructures and Bioimaging, National Research Council (IBB-CNR), Via Pietro Castellino 111, 80131 Naples, Italy
* Correspondence: carla.isernia@unicampania.it

Supplementary Materials

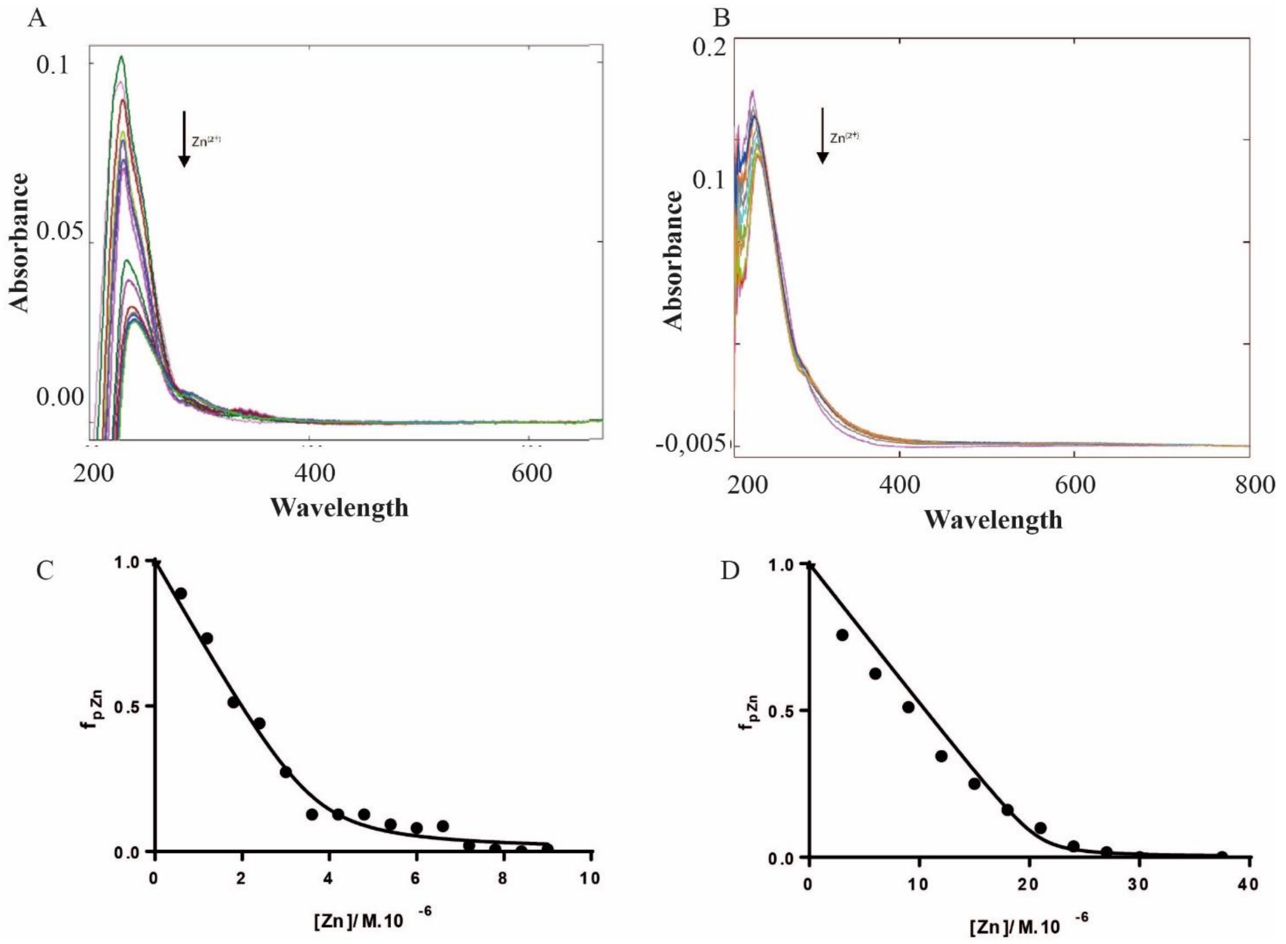

Figure S1. Titration of of Cu(I)-Ros87(**A**) and Cu(II)-Ros87 (**B**) with Zinc ; fitting curves for (**C**) Cu(I)-Ros87 and (**D**) Cu(II)-Ros87.

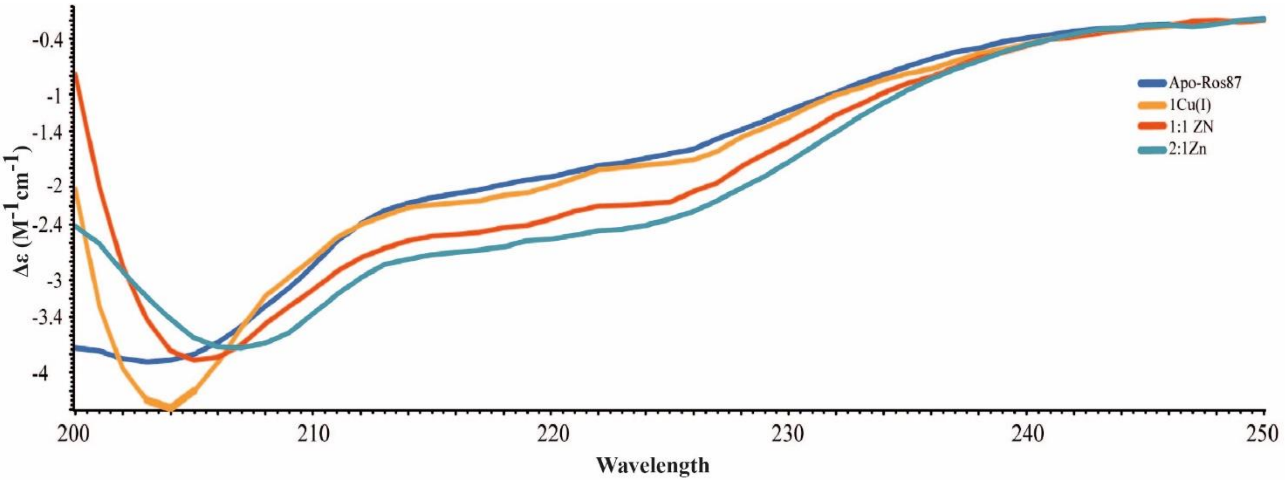

Figure S2. Far-UV CD spectra of Apo-Ros87 (blue line), Cu(I)-Ros87 1:1 (orange), Zn(II)-Ros87 1:1 (red), Zn(II)-Ros87 2:1 (light blue). All the spectra were acquired at 298K.

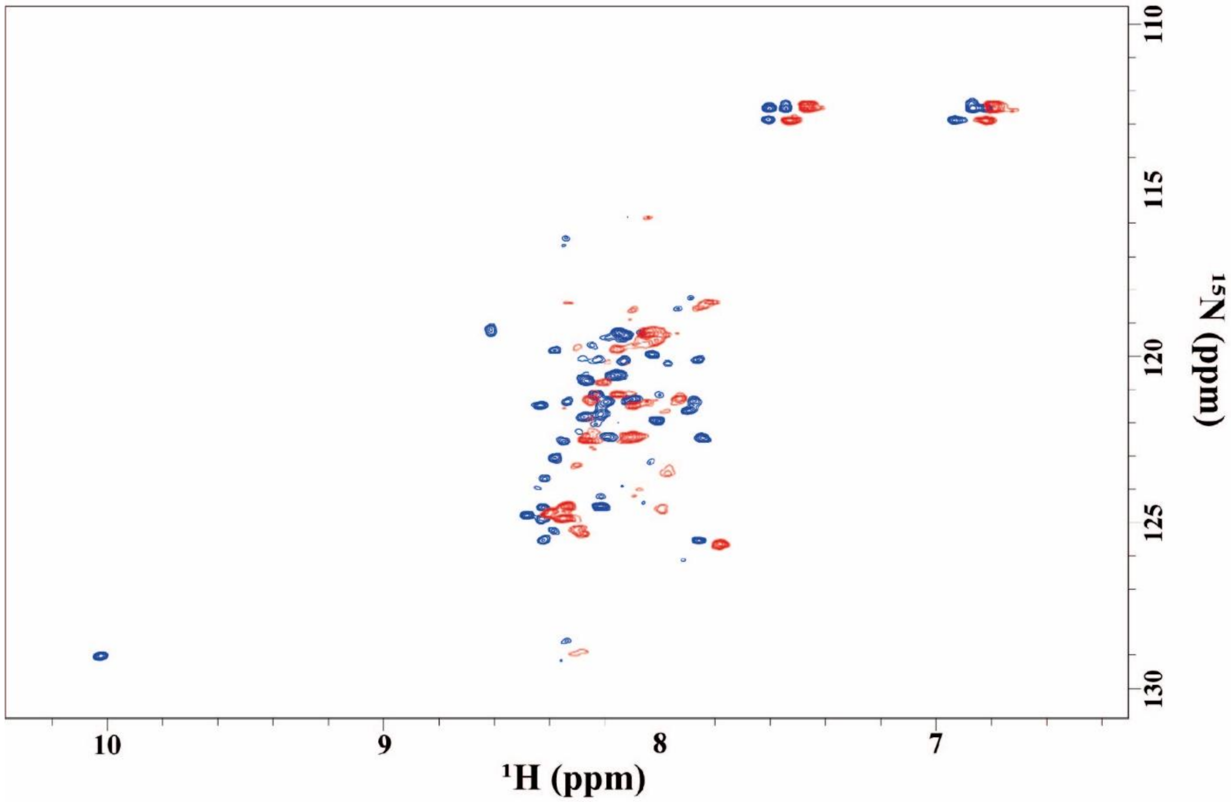

Figure S3. Overlay of the 1H–15N HSQC spectrum acquired for ApoRos87 (blue) with Cu(II)-Ros87 (red).
